# Supplementary material for: Confinement by Liquid‐Liquid Interface Replicates In Vivo Neutrophil Deformations and Elicits Bleb‐Based Migration
Source: Adv Sci (Weinh). 2025 Mar 28;12(21):2414024. doi: 10.1002/advs.202414024 (PMC12140350; doi:10.1002/advs.202414024)
Supplement: Supplementary file 1 — Supporting Information [file ADVS-12-2414024-s005.docx]

Supporting Information

**Confinement by liquid-liquid interface replicates *in vivo* neutrophil deformations and elicits bleb-based migration**

Jonathan H. Schrope^1,2,3,4^, Adam Horn^2^, Kaitlyn Lazorchak^2,4^ , Clyde W. Tinnen^3^, Jack J Stevens^1,2^, Mehtab Farooqui^3,5^,Tanner Robertson^2^, Jiayi Li^1^, David Bennin^5^, Terry Juang^1,3^, Adeel Ahmed^5^, Chao Li^5,7,^**,* Anna Huttenlocher*^2,6,7,^**,  David J Beebe^1,3,5,7,^*

^1^Department of Biomedical Engineering, University of Wisconsin-Madison, Madison, WI, USA.

^2^Department of Medical Microbiology and Immunology, University of Wisconsin-Madison, Madison, WI, USA.

^3^Department of Pathology and Laboratory Medicine, University of Wisconsin-Madison, Madison, WI, USA.

^4^Medical Scientist Training Program, University of Wisconsin-Madison, Madison, WI, USA.

^5^Carbone Cancer Center, University of Wisconsin-Madison, Madison, WI, USA.

^6^Department of Pediatrics, University of Wisconsin-Madison, Madison, WI, USA.

^7^These authors contributed equally.

* Corresponding authors

E-mail: [chaoli3@andrew.cmu.edu](mailto:chaoli3@andrew.cmu.edu); huttenlocher@wisc.edu; djbeebe@wisc.edu

**5. Supporting Information**


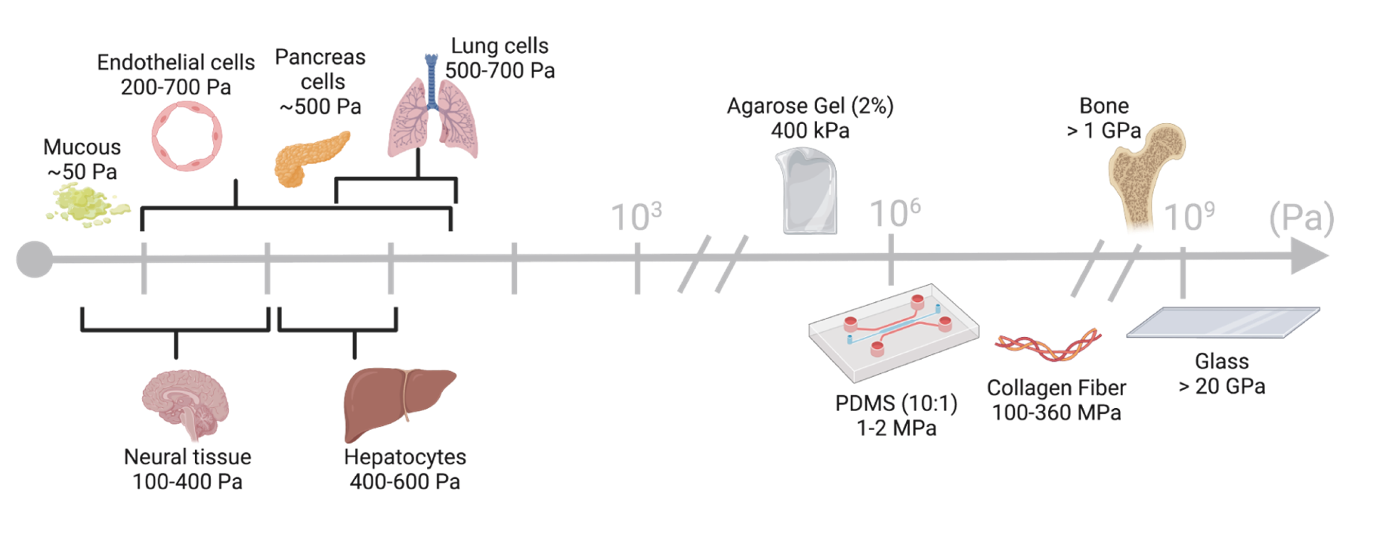


**Figure S1. Review of elastic moduli of individual cells.** Commonly used in vitro materials exhibit elastic moduli orders of magnitude higher than that of single cells. Stiffness values depicted here gathered from a review by Guimarães et al that compiled studies reporting elastic moduli of single cells [65].


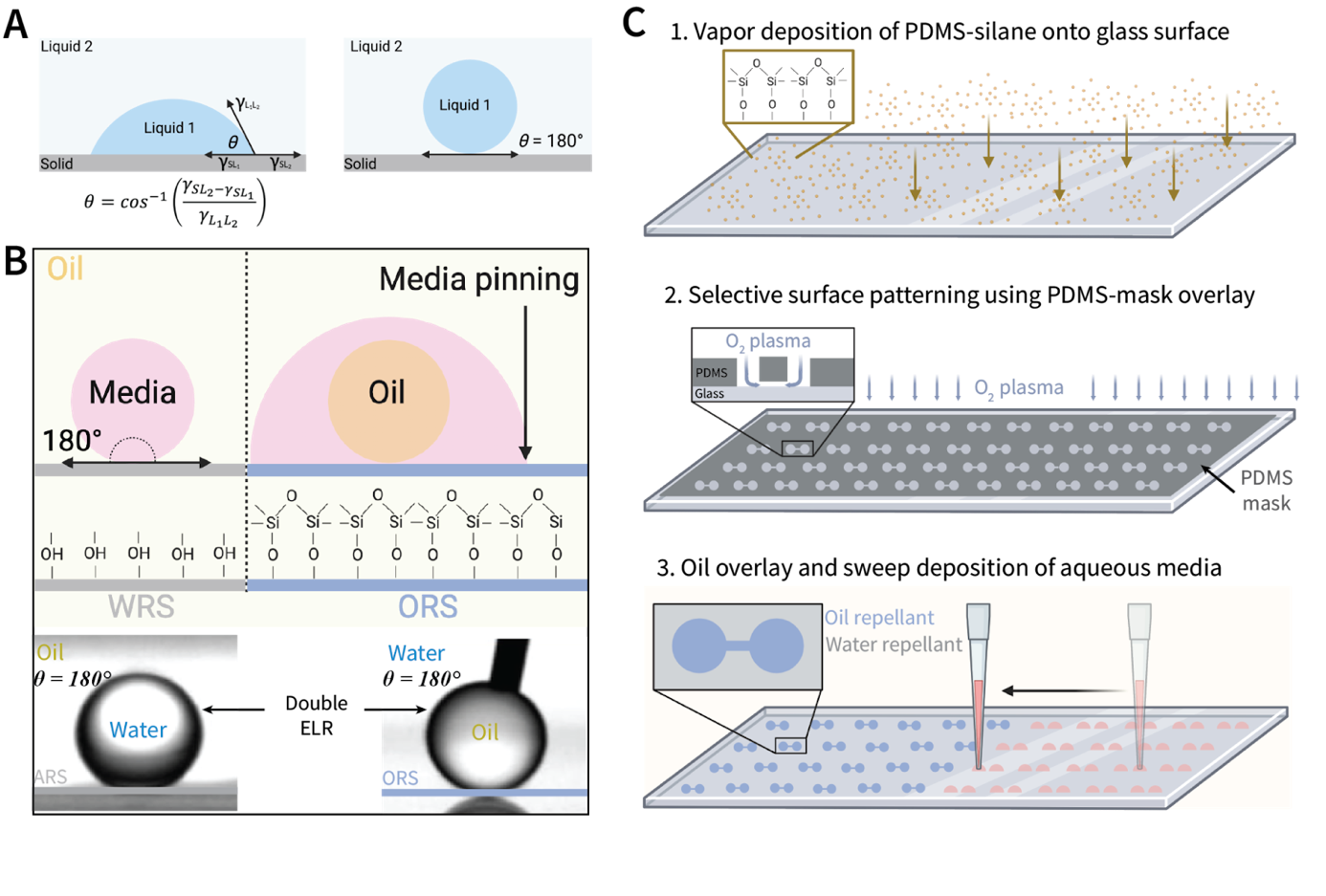


**Figure S2. Construction of liquid microchannels by selective patterning of a glass surface to exhibit double ELR.** A) The degree to which a solid surface repels a liquid within a solid-liquid-liquid three-phase system is reflected in the Young’s contact angle (θ) that a liquid droplet (Liquid 1) makes on the surface. The Young’s contact angle is determined by the balance between all interfacial energies (γSL1, γSL2, γL1L2) present within the system. Precise engineering of this energy balance can result in a surface exhibiting Exclusive Liquid Repellency (ELR), characterized by a Young’s contact angle of 180°. B) Selective chemical patterning of a glass surface to exhibit double ELR results in regions ELR to aqueous media in the presence of oil (Aqueous Repellent Surface, ARS) or ELR to oil in the presence of media (Oil Repellent Surface, ORS). C) Differential surface pattering is obtained by grafting liquid PDMS-silane onto a virgin glass surface by vapor deposition to generate a homogenous ARS surface (repellent to aqueous media in the presence of an oil overlay). A PDMS-based mask is placed on top of the slide and treated with O_2_ plasma. Surface treatment is etched away in areas exposed to O_2_ plasma, where non-exposed areas remain ELR to aqueous media. The mask is removed, and the surface overlaid with oil to generate a double ELR surface, that is, regions repellent to aqueous media (ARS) and regions repellent to oil (ORS).


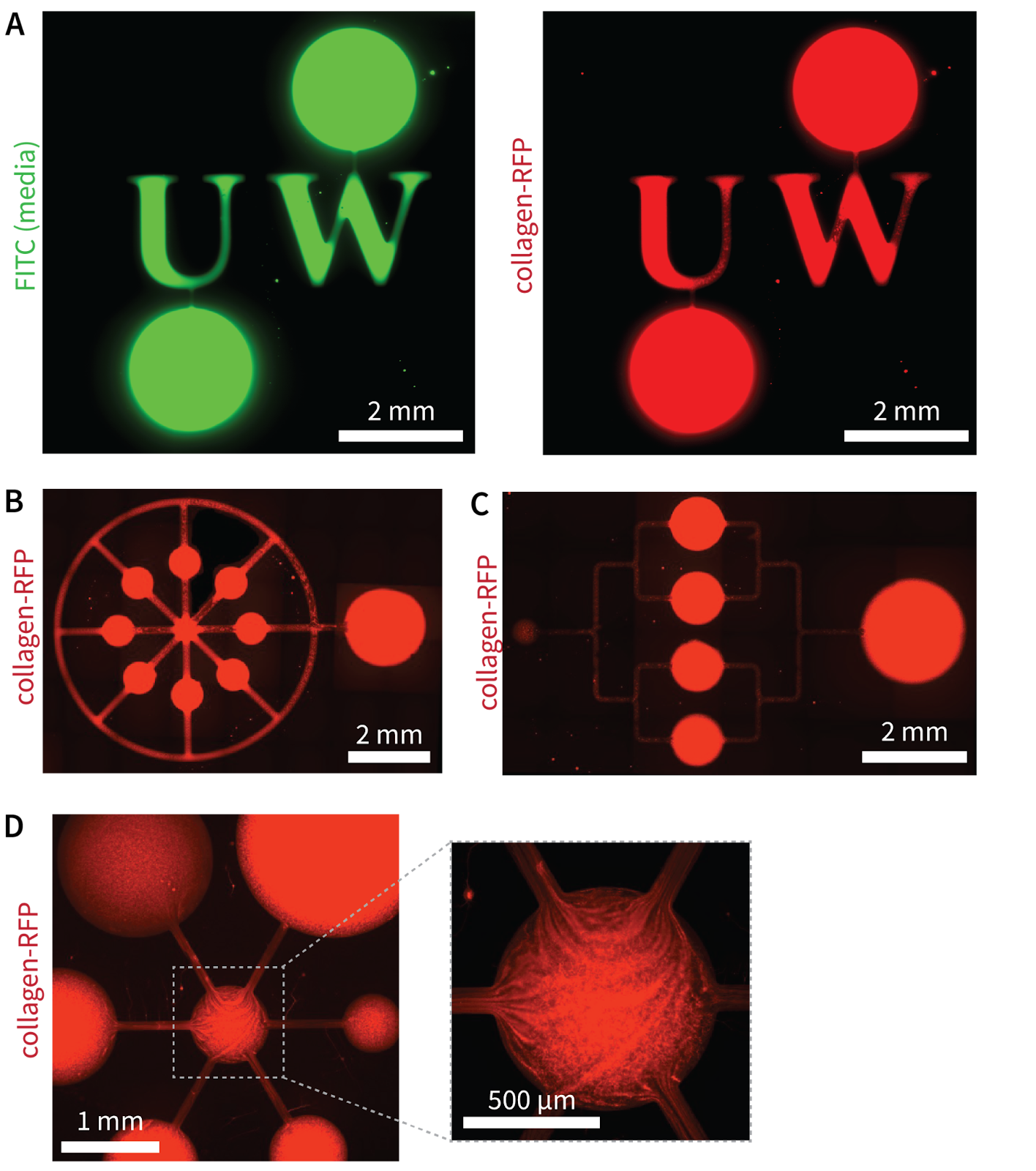


**Figure S3. Examples of different patterns of liquid channels.** A) Representative images of the letters “UW.” Left shows an image of aqueous media labeled with FITC dye and right shows the same pattern made of RFP-labeled fibrous collagen. B-D) Different patterns of collagen channels.


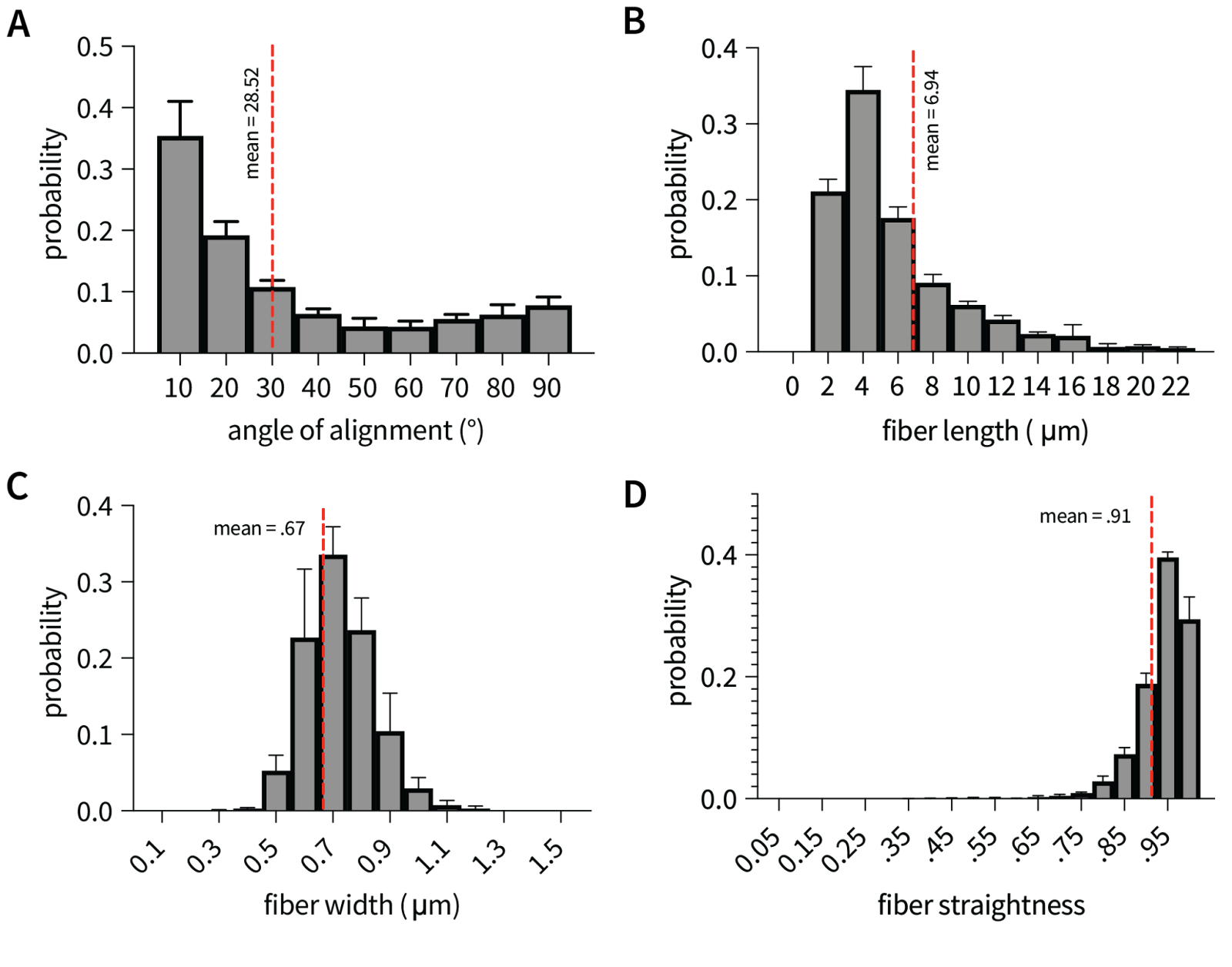


**Figure S4. Characteristics of collagen coating.** The mean A) angle of alignment B) fiber length C) fiber width and D) fiber straightness were calculated over 8 channels (30 µm width) analyzed using CurveAlign software (developed by LOCI at UW-Madison). Notably, collagen structure was not altered during nor after neutrophil migration (data not shown).


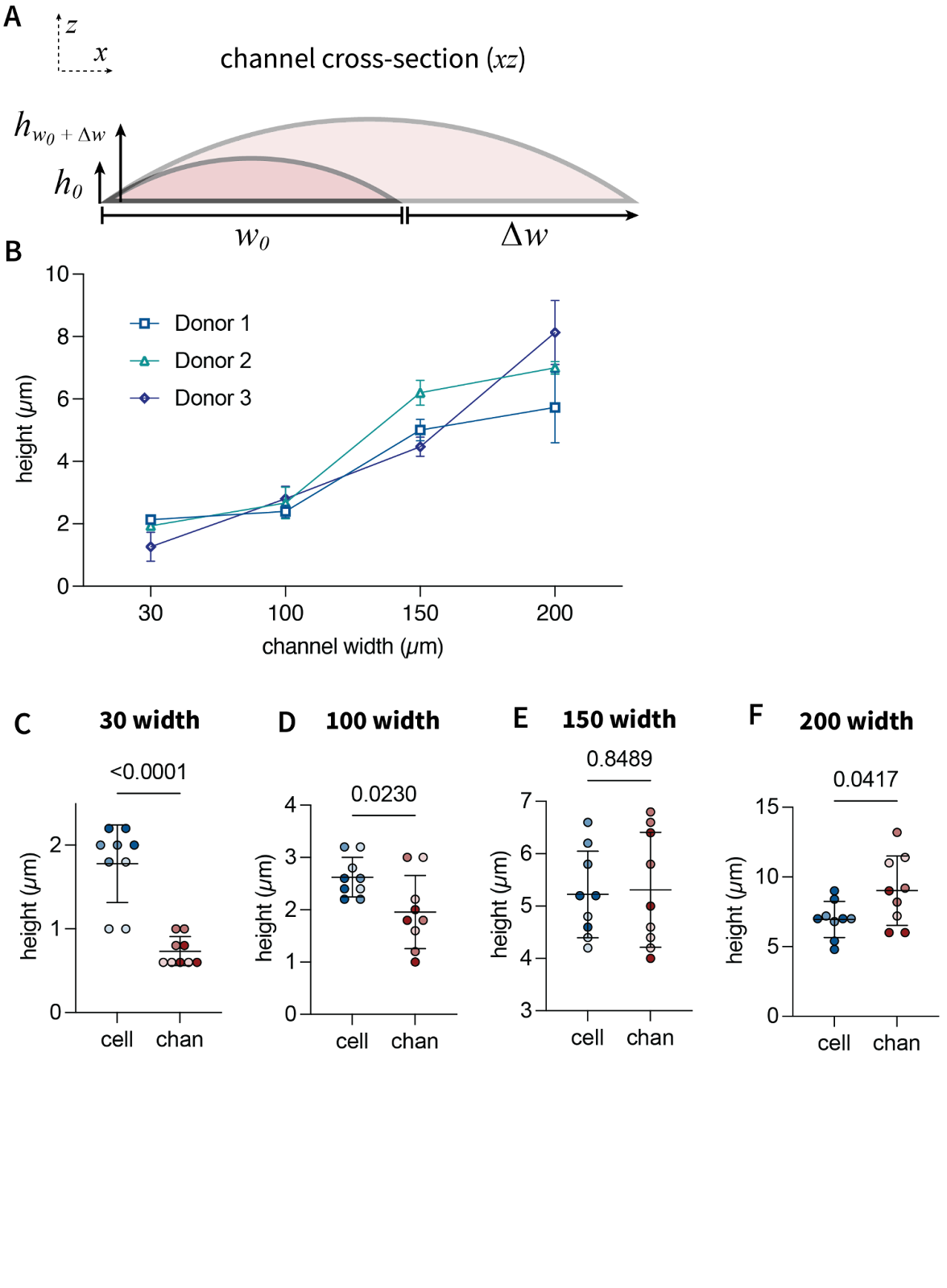


**Figure S5. Channel and cell heights following direct placement of immobile cells into channels.** A) Schematic depicting how increasing channel width increases height, measured as the maximum height at the center of the channel. B) Plot of cell heights including donor variability following direct incorporation into channels of varying width by sweep technique. Each point represents the mean height of a cell (n = 3) measured on independent channel replicates (n = 3) for each donor. C-F) Plots comparing cell height to channel height (in areas absent of cells) on channels of varying width. On small channels with heights significantly less than that of cells (30 and 100 µm width), cells deform the interface to generate height greater than initial channel heights. On large channels (i.e., 200 µm width), cells assume a natural (i.e., non-confined) diameter less than that of the channel height. Each point represents a single cell, shade represents independent donor (n = 3). Statistical significance was determined by an unpaired t-test assuming equal variance.


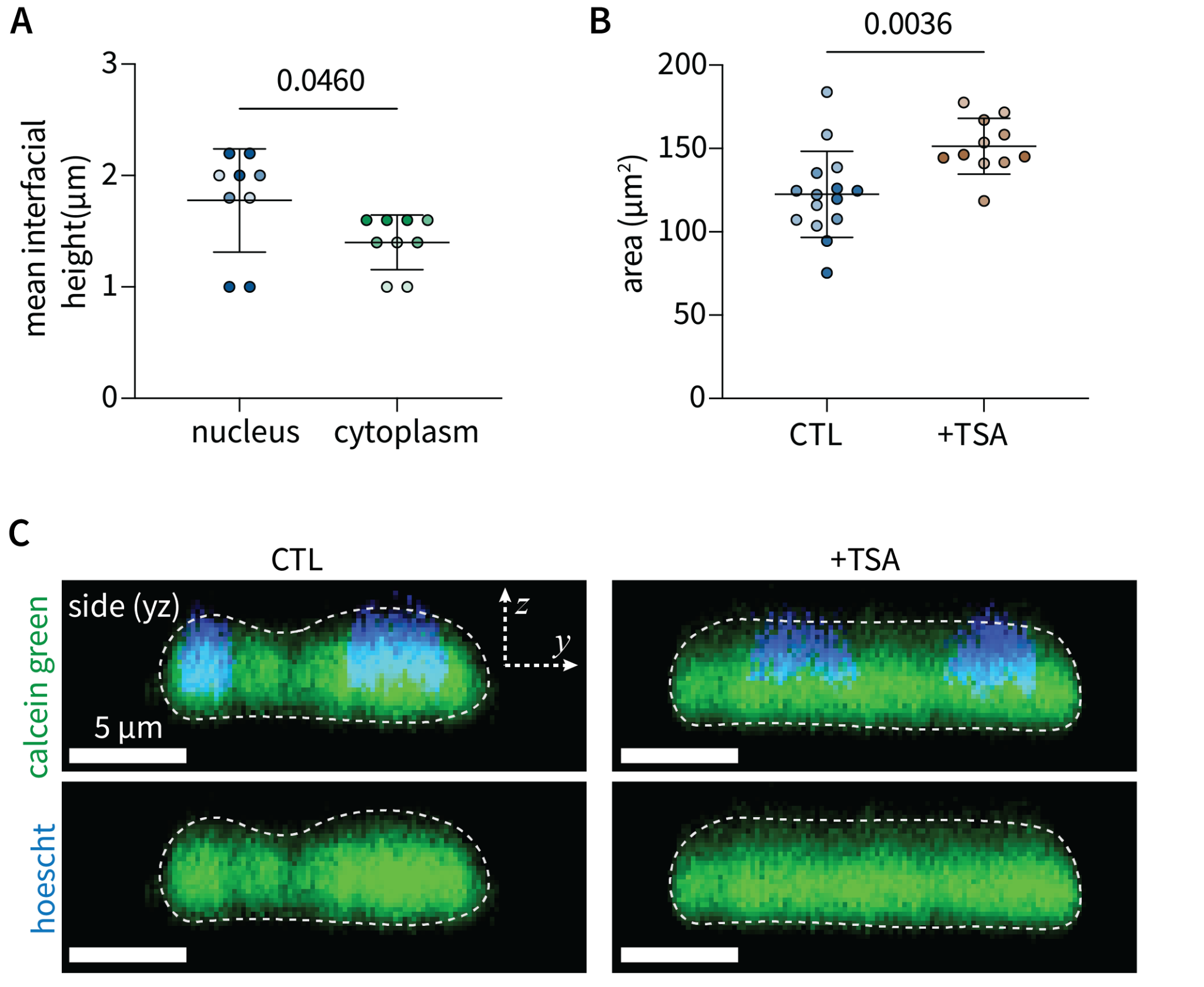


**Figure S6. The neutrophil nucleus determines cell height under confinement by a soft interface.** A) On 100 µm width channels which confine primary neutrophils, cell height is greatest over regions containing nuclear content compared to that of only cytoplasm. Each point represents the mean of cell height (minimum of 3 cells) on an individual channel replicate (n = 3), shade represents independent donor (n = 3). Statistical significance was determined by an unpaired, two-sample t-test assuming equal variance. B) Cell area increases upon treatment with Trichostatin A (TSA) to induce nuclear softening. Each point represents the mean of cell height (minimum of 3 cells) on an individual channel replicate (n = 3), shade represents independent donor (n = 3). Statistical significance was determined by an unpaired, two-sample t-test assuming equal variance. C) Representative images depicting the effect of nuclear softening on cell confinement. Untreated cells exhibit greater height over regions containing nuclear lobes, however under treatment with TSA, cells adopt a flat apical surface independent of nuclear positioning. All experiments were performed on 100 µm width channels.

**The liquid-liquid interface is sufficiently rigid to control cell positioning within a chemical gradient**

As neutrophils mechanically interact with surrounding cells in the body, chemokine gradients arising from sites of injury or infection regulate motility to sites of inflammation. We therefore sought to generate a chemotaxis assay under cell confinement by a liquid interface, which would require spatially patterning of cells within a chemokine gradient. Such gradient generation and control of cell positioning (i.e., “cell trapping”) is commonly achieved using rigid materials that neither cells nor chemokine can permeate [61–63]. Given that the liquid-liquid interface exerts pressures sufficient to confine cells, we hypothesized that this interface could act as a physical barrier to spatially trap cells at the channel entrance within a chemical gradient emanating from the outlet droplet. Thus, channels were constructed with collagen, followed by sequential addition of cells to the inlet droplet by direct pipetting (Figure S7A). The result is a layer of media upon a layer of fibrillar collagen (Figure S7B). If the oil-media interface possesses sufficient rigidity to spatially trap cells within the media layer, then the media layer height would control cell positioning along the channel entrance and thereby enable control of positioning within the chemokine gradient. We find that cells become spatially trapped at the entrance to the channel where interfacial height is comparable to neutrophil diameter (~ 6-12 µm [64]) (Figure S7C). Addition of FITC dye to the outlet droplet establishes a chemical gradient that is stable over experimental timescales (>3 hours) (Figure S7D-E). Taken together, these results demonstrate a novel method of cell trapping within open microscale systems using a liquid-liquid interfacial barrier to control cell positioning within a chemical gradient to study chemotaxis under confinement.


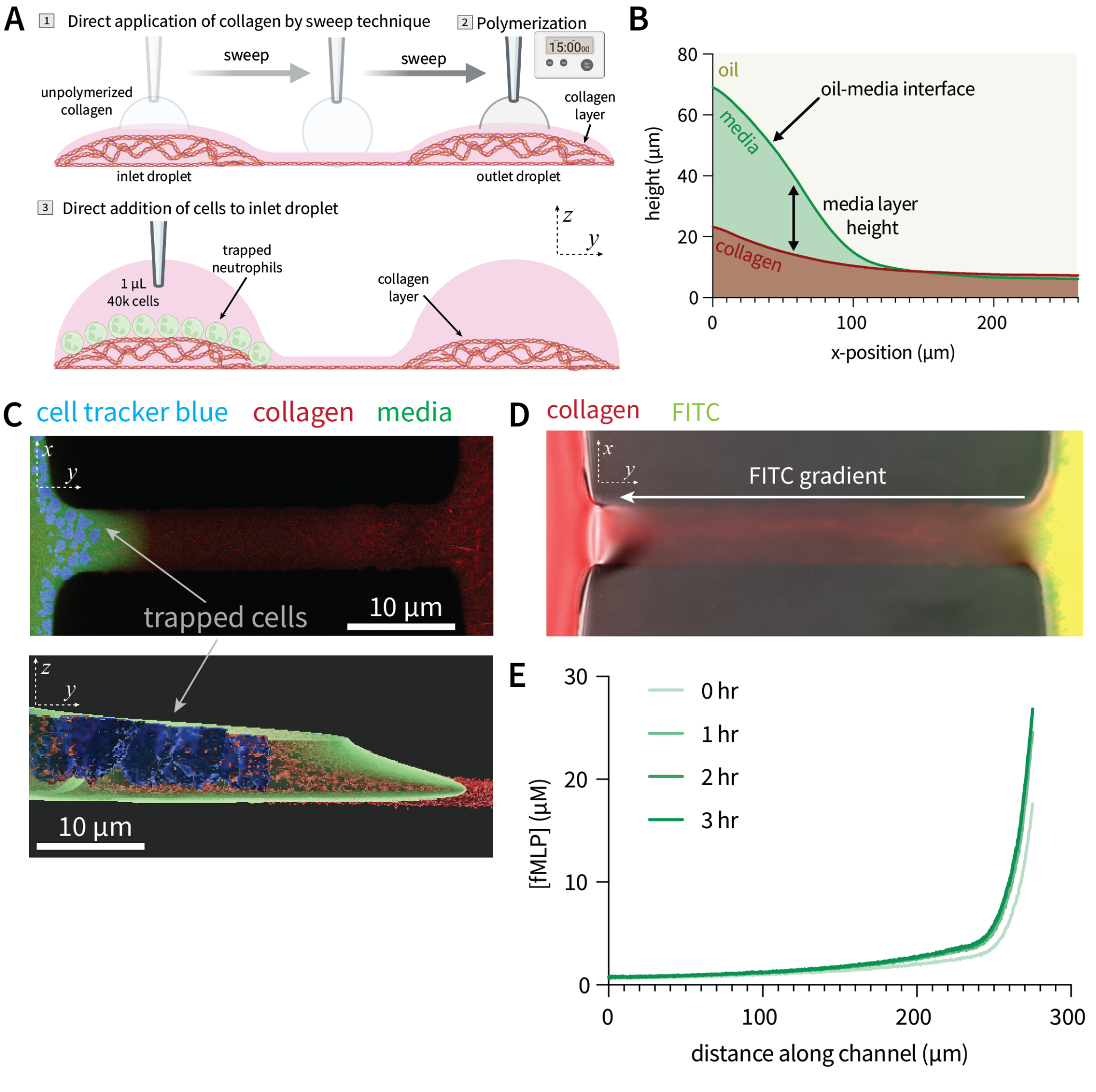


**Figure S7. Single cell trapping and gradient formation within liquid channels.** A) Schematic depicting cell trapping assay whereby cells are added to the inlet droplet following creation of collagen-coated channels. B) Representative profile of the media and collagen layers for a 200 µm width channel. The height of the media layer converges to zero at some point along the length of the channel (~ 130 µm for a 200 µm width channel). C) Representative confocal top and side (Imaris reconstruction) view of cells at the entrance of a 30 µm width and 300 µm length channel spatially trapped at the entrance where media layer height is comparable to cell height (~ 8 µm for neutrophils). D-E) Addition of FITC to the outlet droplet establishes a chemical gradient that is relatively stable over experimental timescales of ~ 3 hours. Approximate concentration of fMLP along channel length was extrapolated from measurements of FITC gradients over time (similar molecular weights of FITC 389.382 g/mol vs fMLP 437.56 g/mol)


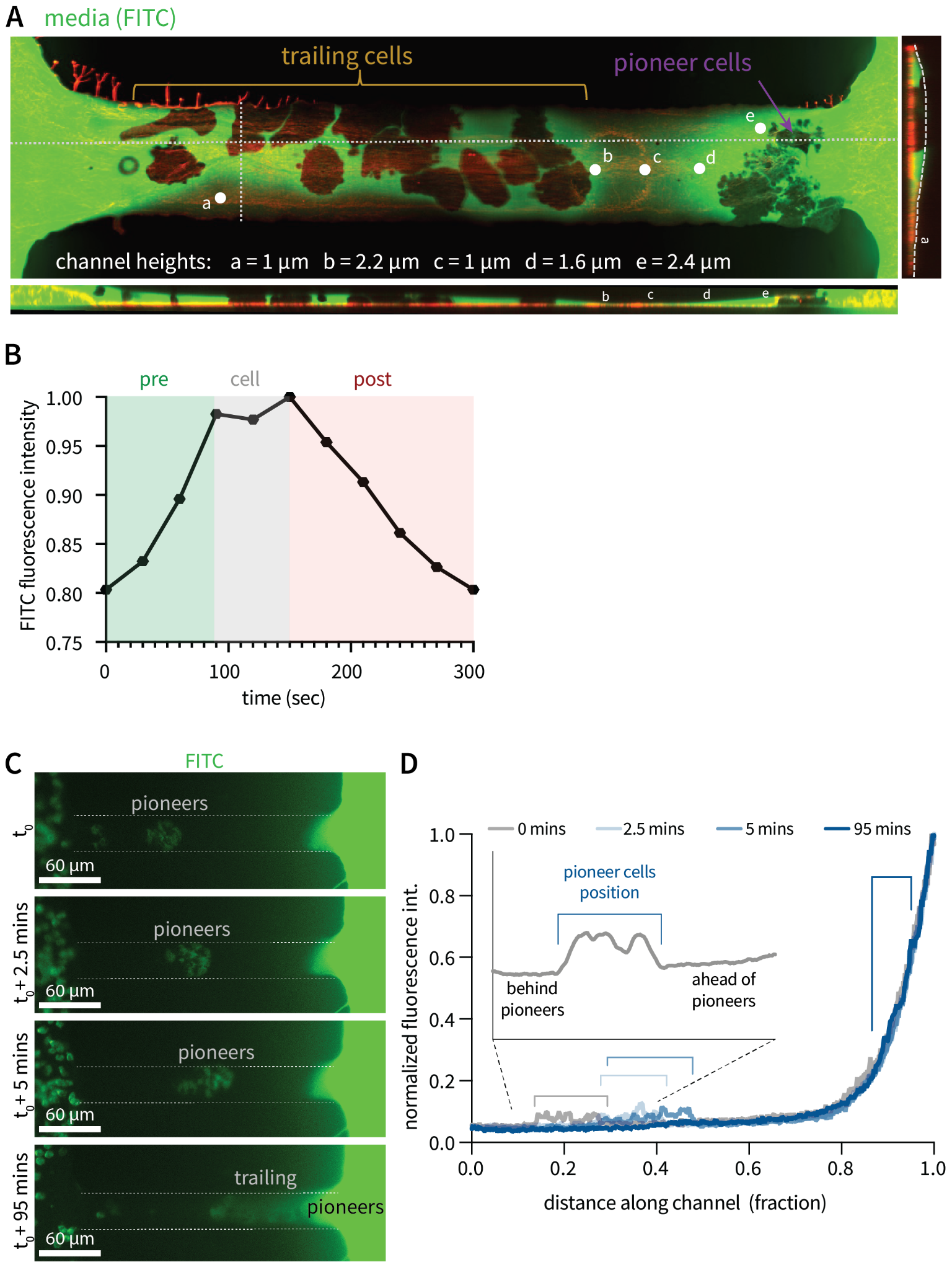


**Figure S8. Interfacial elasticity and gradient stability during neutrophil migration.** A) Representative confocal image of migratory primary neutrophils under confinement by the liquid-liquid interface (30 µm width). Addition of FITC dye added in equal concentrations (40 µM) to the inlet and outlet droplets enables tracking of interfacial height. Notably, the interface relaxes in height in the rare case that there is sufficient distance between cells (point c). B) Representative example of dynamic interfacial deformation and relaxation during pioneer cell passage with FITC added to both the inlet and outlet droplets. FITC intensity was tracked before (pre), during (cell), and after (post) passage of pioneer cells. C) Representative timelapse images of gradient characterization during cell migration with FITC dye added to the outlet droplet. B) Under this method, cells are partially labeled by FITC to mark the position of pioneer cells within the channel. The FITC gradient in front of, or behind the cells is unperturbed over time.


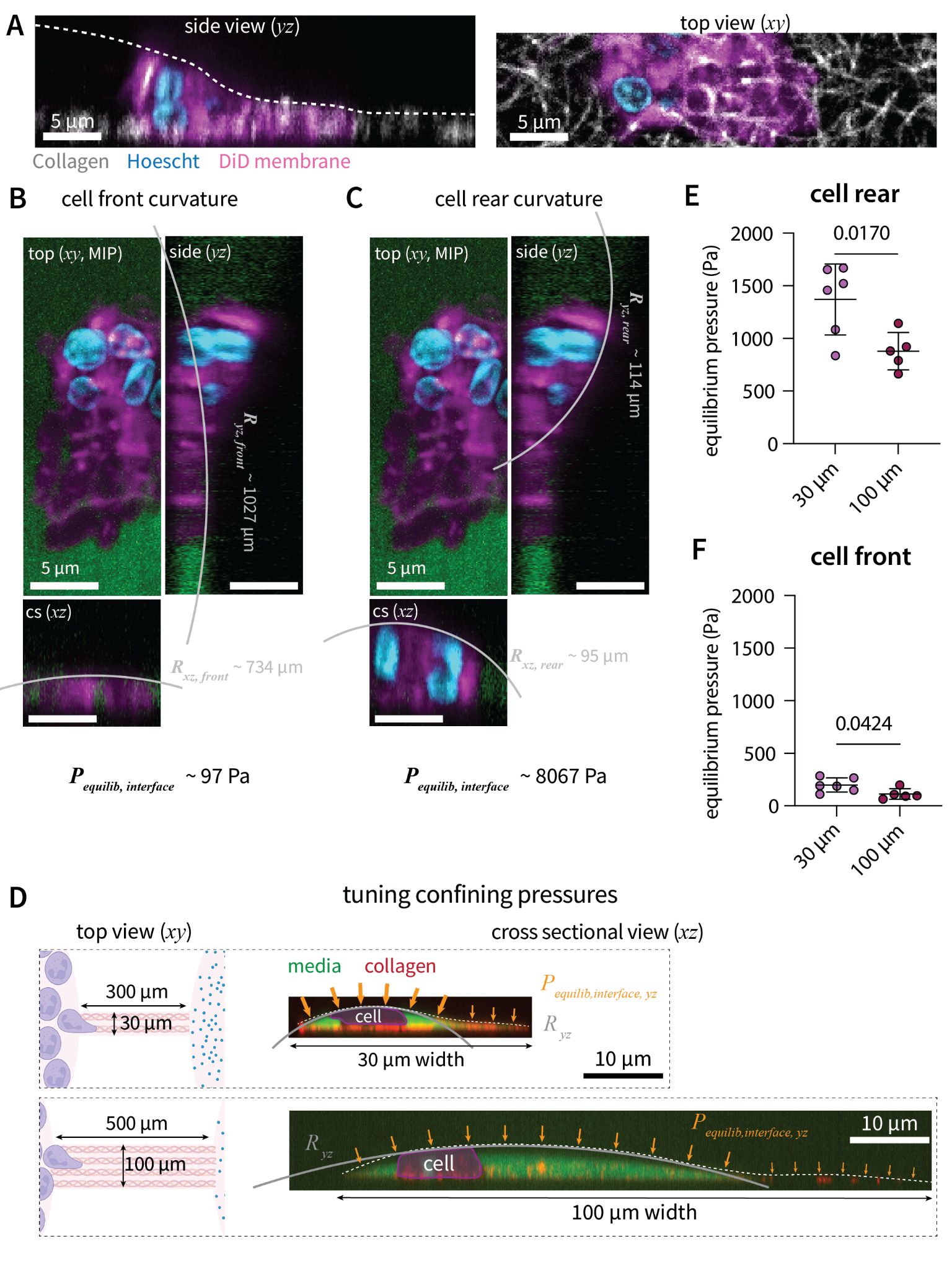


**Figure S9. Pressure calculations and cell morphology as a function of channel width.** A) Confocal side and top view of a representative pioneer cell on a 100 µm channel width on a collagen substrate (gray). B-C) Representative confocal images depicting calculation of curvature in both the *xz* and *yz* planes at the cell front (B) and rear (C). Circles were manually drawn to calculate radii of curvature at different regions along the cell (arcs representative of entire circles are depicted on images). D) Schematic and cross-sectional views of cell height on channels of differential geometry. E-F) Quantification of equilibrium confining pressures over the rear (E) and front (F) of migratory cells within channels of different width. Statistical significance determined by an unpaired, two-sample t-test assuming equal variance.


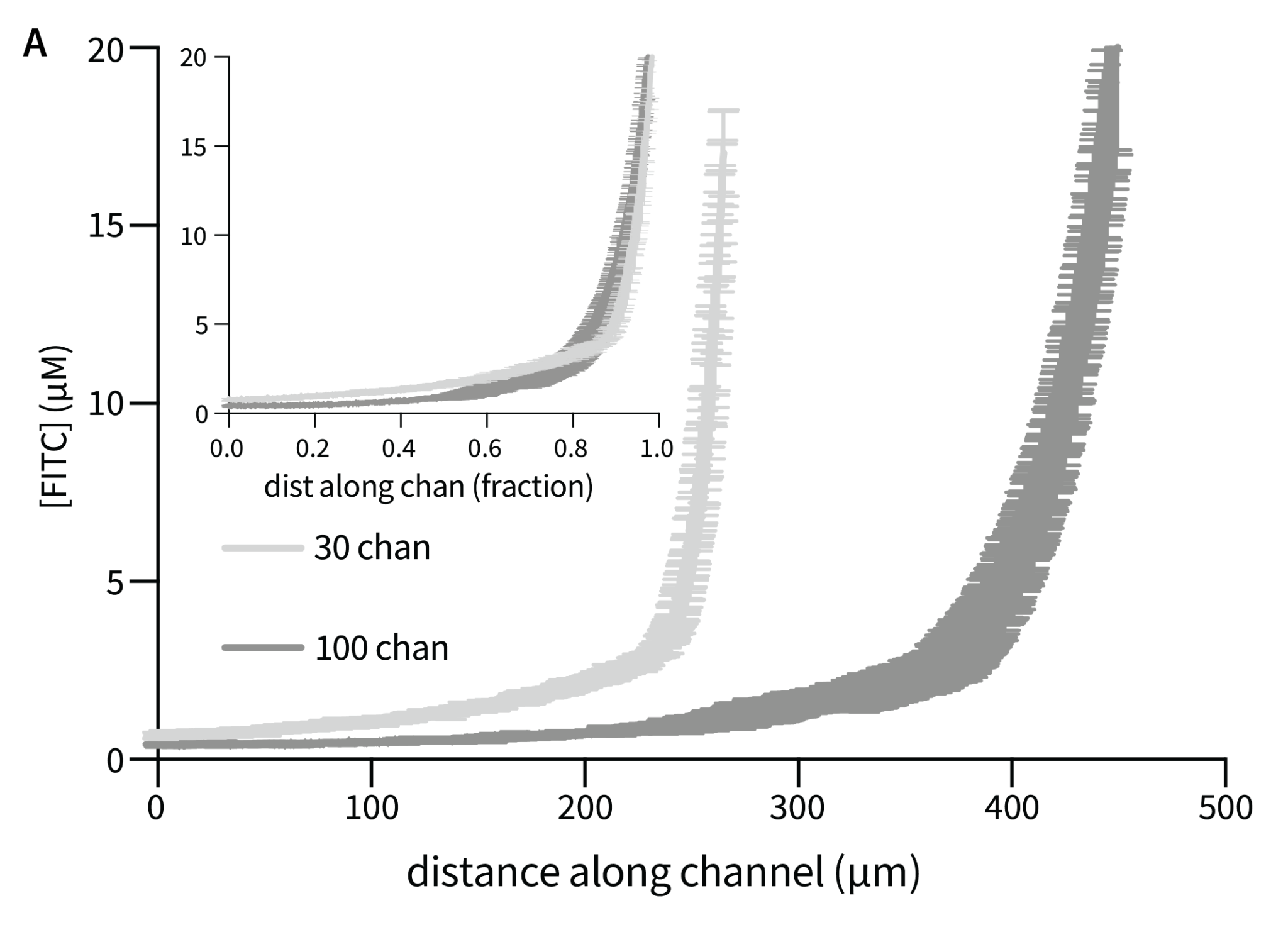


**Figure S10. Tuning channel geometries does not significantly alter the chemokine gradient.** A) Addition of FITC to the outlet droplet only enables estimation of the fMLP gradient during cell migration (as in Fig S8C-D). The 30 µm width 300 µm length channel yields a slightly steeper gradient which would hypothetically yield faster migration (our data indicates slower, attributed to differences in mechanical resistance). Notably, all migration data in the manuscript was analyzed up to .8 fractional along the channel, before cells reach the region where the interface curves upward to meet the outlet droplet (where significantly increased height of the outlet droplet increases fluorescence intensity).


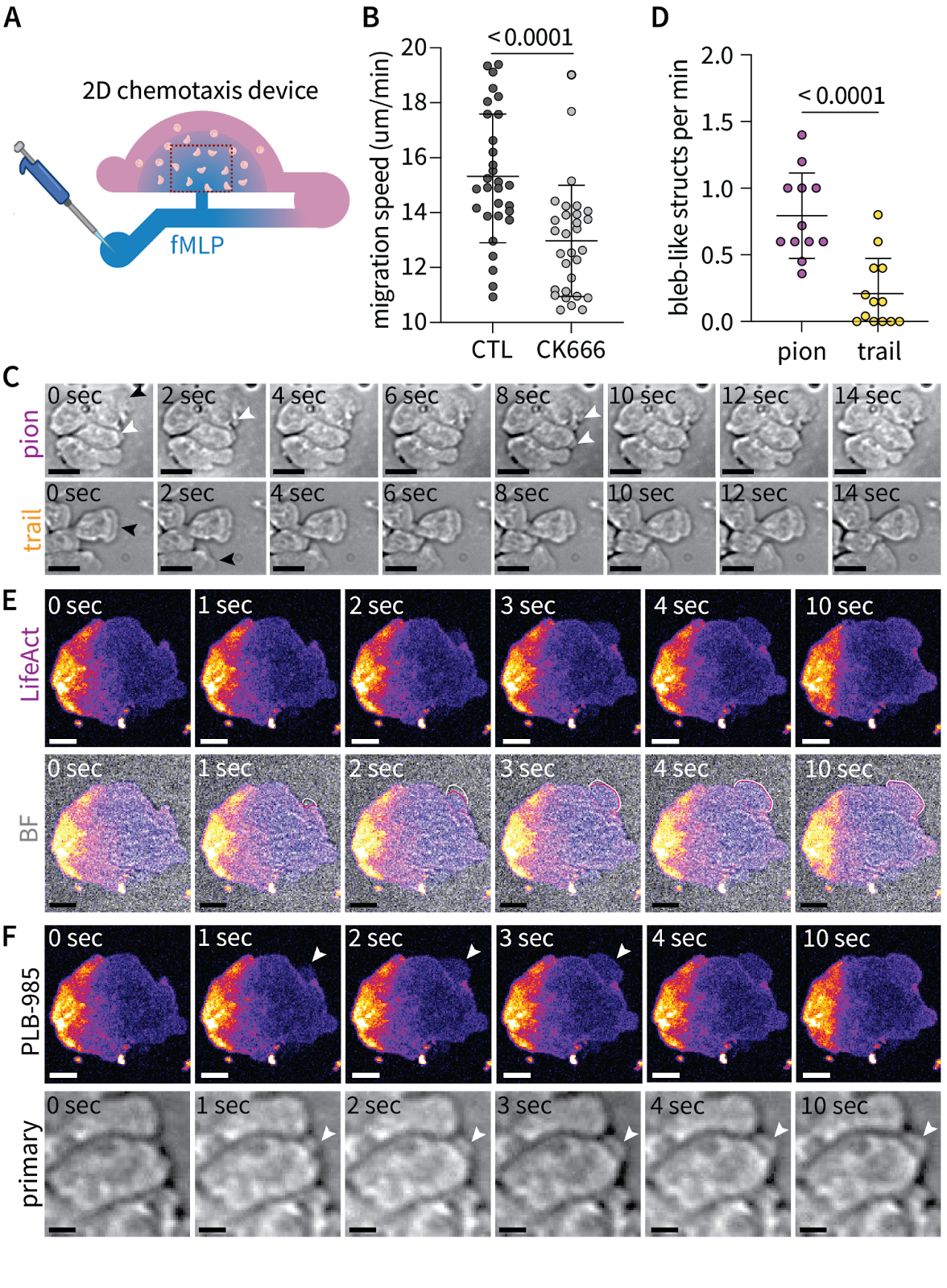
**Figure S11. Characterization of primary neutrophil and neutrophil-like PLB-985 cell migration mode.** A) Schematic of two-dimensional chemotaxis device B) Pioneer cell migration speed in the absence of confinement is decreased under treatment with the Arp-2/3 inhibitor CK666. Significance was determined by an independent two sample t-test between the two experimental groups (n = 30 cells each, 10 tracked over 3 device replicates) C) Brightfield time lapse images of pioneer and trailing cell migration. White arrows indicate bleb-like structures and black arrows indicate sheet-like pseudopodal protrusions. Scale bar represents 10 µm. D) Quantification of bleb-like structures per minute for pioneer and trailing primary neutrophils. Circles represent individual cells, data pooled evenly over three independent donor replicates with three independent channels per donor replicate. E) Timelapse images of LifeAct-mRuby expressing neutrophil-like PLB-985 cells depicting membrane extension (white line) past actin signal (red line) at initial stages of bleb formation. Scale bars represent 5 µm. F) Timelapse images depicting similar shape and formation kinetics between bleb-like structures of primary neutrophils and blebs of neutrophil-like PLB-985 cells. Scale bars represent 5 µm.

**6. Supporting Information Movie Legends**

**Movie S1 (separate file). Liquid Channels.** Three-dimensional artistic rendering of collagen-coated liquid channels located between two media droplets (inlet and outlet). Mesh denotes oil-media interface.

**Movie S2 (separate file). Pioneer cell morphology.** Three-dimensional confocal reconstruction of a pioneer cell during migration, generated using Icy image analysis software.

**Movie S3 (separate file). In vivo neutrophil interstitial migration.** Timelapse of neutrophil interstitial migration depicting deformations of surrounding basal keratinocyte cells (green) during the protrusion stage and passage of the cell body (blue; cytoplasm) containing the nucleus (red). Time given as mm:ss.

**Movie S4 (separate file).** Pioneer and trailing primary neutrophil migration. Timelapse depicting migration of pioneer (left) and trailing (right) primary neutrophils. White arrows depict rapid bleb-like protrusions and black arrows sheet-like pseudopodal protrusions. Images taken at an interval of 0.5 sec, time given as mm:ss.

**Movie S5 (separate file). Trailing PLB-985 cell actin dynamics.** Timelapse depicting sheet-like protrusions of a trailing PLB-985 cell expressing LifeAct-mRuby migrating within a 30 µm width channel.

**Movie S6 (separate file). Pioneer PLB-985 cell actin dynamics.** Timelapse depicting bleb protrusions of a pioneer PLB-985 cell expressing LifeAct-mRuby migrating within a 30 µm width channel.

**Movie S7 (separate file). Transition to blebbing.** Timelapse depicting cell transition from sheet-like pseudopodia to blebs upon reaching the interface in LifeAct-mRuby expressing PLB-985 cells within 30 µm width liquid channels. Movie contains a pioneer cell (top) and a trailing cell (bottom) that migrates fast enough to reach the interface and transition to bleb protrusions at the leading edge. Images taken at 1 sec intervals.

**Supporting Information References**

61. X. Wang, Z. Liu, Y. Pang, Concentration gradient generation methods based on microfluidic systems. RSC Adv. 7, 29966–29984 (2017).

62. H. Somaweera, A. Ibraguimov, D. Pappas, A review of chemical gradient systems for cell analysis. Anal Chim Acta 907, 7–17 (2016).

63. V. V. Abhyankar, M. A. Lokuta, A. Huttenlocher, D. J. Beebe, Characterization of a membrane-based gradient generator for use in cell-signaling studies. Lab Chip 6, 389–393 (2006).

64. P. X. Liew, P. Kubes, The Neutrophil’s Role During Health and Disease. Physiol Rev 99, 1223–1248 (2019).

65. C. F. Guimarães, L. Gasperini, A. P. Marques, R. L. Reis, The stiffness of living tissues and its implications for tissue engineering. Nature Reviews Materials 5, 351–370 (2020).
